# Squatting, pelvic morphology and a reconsideration of childbirth difficulties

**DOI:** 10.1093/emph/eoac017

**Published:** 2022-04-26

**Authors:** John Gorman, Charlotte A Roberts, Sally Newsham, Gillian R Bentley

**Affiliations:** 1 Independent Scholar, Greenhead, Brampton, Northumberland CA8 7HX, UK; 2 Department of Archaeology, Durham University, Dawson Building, South Road, Durham DH1 3LE, UK; 3 Department of Gynaecology, North Cumbria Integrated Care NHS Foundation Trust, Cumbria CA2 7HY, UK; 4 Department of Anthropology, Durham University, Dawson Building, South Road, Durham DH1 3LE, UK

**Keywords:** squatting, childbirth, obstetrical dilemma, pelvic morphology, first epidemiological transition

## Abstract

Childbirth is commonly viewed as difficult in human females, encompassed by the ‘Obstetrical Dilemma’ (OD) described by early palaeoanthropologists as an evolved trade-off between a narrow pelvis necessitated by bipedalism and a large-brained fetal head. The OD has been challenged on several grounds. We add to these challenges by suggesting humans likely squatted regularly during routine tasks prior to the advent of farming societies and use of seats. We suggest that habitual squatting, together with taller stature and better nutrition of ancestral hunter-gatherers compared with later Neolithic and industrial counterparts, obviated an OD. Instead, difficulties with parturition may have arisen much later in our history, accompanying permanent settlements, poorer nutrition, greater infectious disease loads and negligible squatting in daily life. We discuss bioarchaeological and contemporary data that support these viewpoints, suggest ways in which this hypothesis might be tested further and consider its implications for obstetrical practice.

**Lay Summary:**

Human childbirth is viewed as universally difficult. Evidence from physical therapies/engineering and studies of living and ancestral humans illustrates habitual squatting widens the pelvis and could improve childbirth outcomes. Obstetrical difficulties emerged late in prehistory accompanying settled agriculture, poorer nutrition and less squatting. Specific physical exercises could improve obstetrical practice.

## THE OBSTETRICAL DILEMMA

Since Washburn’s [1] characterization of human childbirth as the ‘Obstetrical Dilemma’ (OD), researchers in anthropology have grappled with the problem of explaining difficulties during human parturition that can result in high rates of morbidity and mortality for both mothers and infants during delivery [2–5]. The problem is framed by the increasing size of the human brain during hominin evolution coupled with the constraints of bipedality that posed technical difficulties for the process of childbirth. These presumed constraints led Trevathan and Rosenberg to conclude that human birth attendants became virtually obligatory during human evolution to ensure safer birth outcomes [4–7].

Washburn [1] and Lovejoy *et al*. [8] both argued that bipedal walking necessitated a narrower pelvis compared to our earlier quadrupedal ancestors, thus introducing a trade-off for childbirth once larger hominin brains evolved [4, 9]. Details of the mechanical constraints of bipedality have been provided in several papers [3, 10, 11] but, briefly, becoming bipedal altered the center of gravity during walking and both the shape of the spine and pelvis had to adapt to these constraints. Consequently, the lower (lumbar) human spine demonstrates considerable lordosis, the ilia became shortened and the pelvis adopted a rounder or more bowl-like configuration. In relation to obstetrical constraints, the *Homo* pelvis relative to other apes now has an inlet that is wider at the transverse point rather than sagittally, exacerbated by ventral protrusion of the sacral promontory and a narrowing of the mid-pelvis at the transverse plane by medial protrusion of the ischial spines. These are attached to ligaments that support the abdominal viscera, while the antero-posterior diameter of the pelvic outlet is limited at the outlet by protrusion of the sacrum and coccyx [12, 13]. Furthermore, with regard to the pelvic bones, the ilium fuses to the combined ischiopubic portion at the acetabulum between 11 and 15 years in females (and 14–17 years in males) to form the os coxae/innominate bone. However, pelvic growth trajectories in females between puberty and ∼25–30 years provide for larger obstetrically-related dimensions [14, 15]. This allows continued plasticity of the pubic rami throughout the primary childbearing years [16].

Given the breadth of the infant head, coupled with a bipedal pelvis that is widest at the inlet in the medio-lateral plane versus widest in the antero-posterior plane at the outlet, the human baby has to navigate a relatively small passage during parturition and rotates its position twice during the delivery process [17]. At the pelvic inlet, the sagittal diameter of the fetal head is orientated either transversely or obliquely, but when navigating through to the pelvic outlet, the head has to reorientate itself so that the sagittal plane is now aligned sagittally with this pelvic plane. Finally, when the head emerges from the pelvis, the fetal shoulders also have to rotate to align sagittally with the pelvic outlet.

The initial description of the OD has been challenged in recent years as more evidence has emerged concerning the mechanisms of human locomotion and primate maternal investment [18]. At present, there are at least two competing hypotheses to explain constraints on the human pelvis aside from the OD, namely: (i) visceral support and (ii) thermoregulation. With respect to the former, a number of scholars have suggested that, when adopting an obligate, upright position, better support is provided for the abdominal viscera and a heavy fetus at term if the pelvis is relatively narrow [12, 19–22]. Here, the protrusion of the ischial spines are particularly relevant despite making childbirth more complicated [21]. The visceral support hypothesis is likewise upheld by evidence for an increasing number of pelvic floor disorders in women who have a wider pelvic inlet, outlet or pelvic floor area [21, 23].

With regard to the second hypothesis concerning thermoregulation, Ruff has suggested pelvic width, as measured by the intercristal diameter (the distance between the crests of the ilia), is affected by trunk size in humans which, itself, is heavily influenced by Bergmann’s Rule governing thermoregulation [24, 25]. This argues that body proportions in hotter climates tend to be elongated in order to facilitate heat exchange with a higher surface-to-body ratio, and exemplified by many East African pastoralist populations such as the Turkana. The opposite holds true in higher latitudes where people tend to be short and squat (i.e. short legs and heavy musculature), exemplars being the Inuit. Such patterns across latitudes are also reflected in earlier *Homo* species. However, the thermoregulation hypothesis has been challenged by data from Betti *et al*. [26] who demonstrated, using a range of pelvic osteological data from numerous global populations, that pelvic shape has been affected more by genetic drift (neutral evolution as a function of increasing distance from founding populations in southern Africa) than by climate, and that climate has more impact on the femur and tibia [26, 27]. However, Betti *et al*. also found from their series of measurements that the true pelvis (relating to the pelvic midplane and outlet which places greater constraints on the birth process) was likely more subject to natural selection than the false pelvis (relating to the inlet) [26]. These differences, however, were mirrored across both males and females despite the inference that selection might result from obstetrical difficulties.

Aside from these two competing arguments, other aspects of the OD hypothesis have been challenged. For example, the supposed constraints on efficient walking and running in females because of a greater bi-acetabular width have been contested by Warrener *et al*. who conducted experiments to test whether females have less efficient locomotion as a result of their slightly wider pelvis [11, but cf. 28]. Their results did not support any sex differences in either locomotion or hip abductor mechanics, although similar studies of women in the third trimester of pregnancy might be a useful, additional comparison. Second, humans are not unique in having a tight fit between a fetal head and pelvic outlet, nor in requirements for the fetus to rotate during parturition [29]. In relation to gestation length being constrained by the size of the infant brain versus a narrow pelvis, Dunsworth *et al*. [2] have pointed out several flaws in the OD argument. They suggested that gestation length in humans depends more on metabolic constraints than mechanical ones. The authors called this the ‘Energetics of Gestation and Growth Hypothesis’ advanced in earlier form by Ellison [30], and first articulated by Martin [31]. Dunsworth *et al*. [2] also demonstrated that, taking into account maternal body size, human maternal investment is greater than our closest primate relatives, since both infant brains and bodies are larger while gestation length is longer than would be expected for a primate of similar body mass.

In addition, several authors have argued for new approaches to the OD that better consider issues of developmental plasticity. Indeed, the plasticity of the pelvis and the influences of nutritional constraints and types of physical activities on the pelvis during development have been known for some time [32–35]. Many incidences of dystocia (obstructed labor) recorded in lower and middle income countries (LMICs) occur in young mothers and are related to trade-offs between growth and reproduction resulting in compromised development of individuals due to early life nutritional constraints [36–38]. This can result in the pelvis being flattened or otherwise misshapen, thus contributing to cephalopelvic disproportions [36]. Specific micronutrient deficiencies such as vitamin D (from inadequate exposure to sunlight) would have similar effects [39]. All these challenges to the OD have been usefully visualized by Haeusler *et al*. [18] in their Fig. 1.

**Figure 1.**
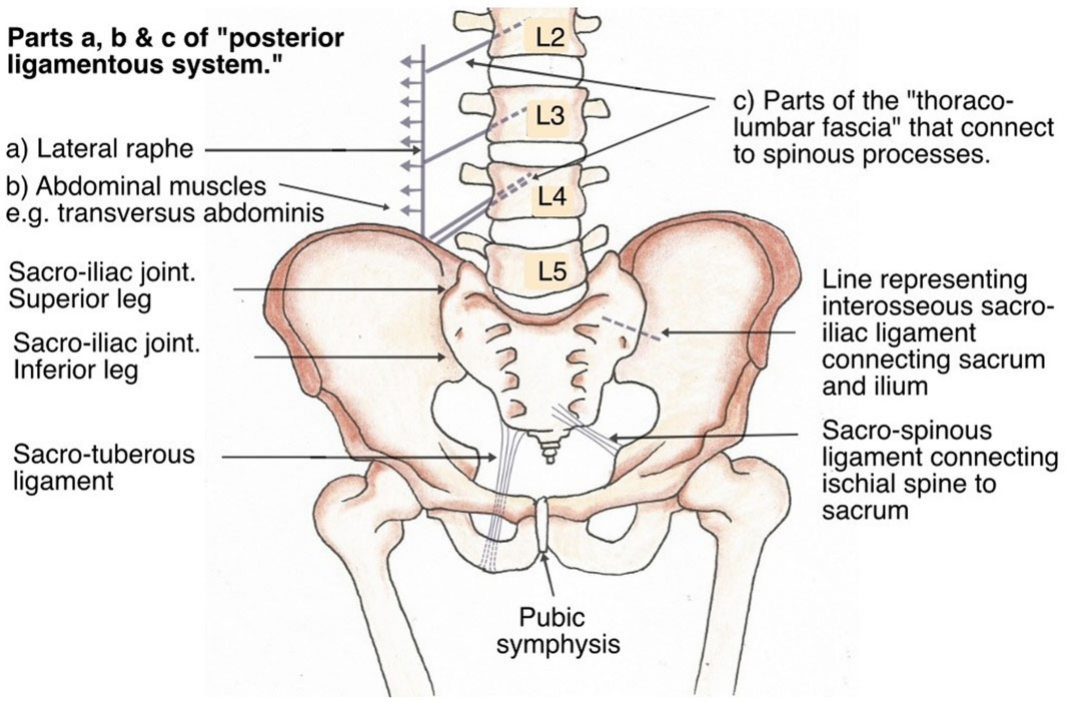
Anterior view of the human pelvis showing the ligaments holding the structure together. Figure drawn by Yvonne Gorman.

## PLASTICITY OF THE PELVIS

Supporting ideas of pelvic plasticity, Driscoll [40], in her dissertation tracing changes in pelvic morphology in more recent US skeletal series dating from 1840 to 1981, found noticeable differences across time that she speculated were related to nutritional improvements as well as decreases in physical activity. Specifically, both the antero-posterior diameter of the pelvic inlet and the transverse diameter of the outlet were becoming more elongated. There was also an increased rounding of the pelvic canal and a decreased flaring of the pelvic bowl [40]. Women’s height is also a strong factor in increasing maternal morbidity risk, with cut-offs varying across different countries and being markedly lower in LMICs [36, 38, 41]. Conversely, in many higher income countries, increasing rates of overweight, obesity and gestational diabetes have led to larger infant sizes, with consequent problems during delivery [34, 42, 43].

In the late 19th century, anatomists had in fact already observed considerable variation in pelvic shape. However, they did not consider the influence of early life development, classifying shapes initially into three types (dolichopellic, mesatipellic and platypellic); later they extended this classification to four types to include brachypellic [44–46]. In the 1930s, Caldwell and Moloy published a series of influential papers that categorized the female pelvis into four primary shapes (platypelloid, gynecoid, anthropoid and android) based on the shape of the pelvic inlet [47–49] (Supplementary Fig. 1). All of these classifications were heavily influenced by ‘racial’ typologies [39, 50], and might justifiably be considered *historic* were they not still reiterated in medical textbooks even to the present day [51–54].

The Caldwell and Moloy typology considered the ‘gynecoid’ or ovoid type as optimal for parturition, while having an anthropoid pelvis (attributed to non-Europeans) was also associated with good birth outcomes. Strangely, the platypelloid or flattened pelvis, now considered a marker of nutritional deficits during growth and associated with problematic births, was thought to belong to more ‘civilized racial’ groups by the progenitors of the typologies [39]. These ‘racial’ associations were challenged later by Greulic *et al*. who pointed out that just over a third were gynecoid in the women studied [55, 56]. More recently, Betti and Manica have confirmed the wide range of variability in shapes of human pelves and birth canals in 348 women sampled across wide geographic areas [57]. They suggested this is due to genetic drift following human migrations out of Africa where variability appears to have been higher. In contrast, Kuliukas *et al*. [51] used computer tomography to characterize the pelves of 64 women from Western Australia concluding that the resulting variation may be due to a variety of epigenetic and developmental factors.

Finally, a number of authors have questioned the historical context in which the OD Hypothesis was framed, pointing out its cultural roots and the belief that women were more frail than men [39, 57–59]. Furthermore, the medicalization of birth over recent centuries should be considered together with the influence of sexual selection theory, and a lack of appreciation of variability in shapes of the human pelvis [39, 58–60], as well as how developmental processes can affect these shapes [32]. Walrath has also indicated that the gynecoid model of the human pelvis and the associated rotational birth pattern have come to dominate both obstetrical and anthropological discourse on the process of birth, leading to a singular rather than variable model of the birthing process with major implications for the medical management of birth today [39]. Just as pelvic shape varies considerably across individuals, so does the likelihood of variation in how infants navigate the birth passage.

Hypotheses aside, several adaptations have arisen to meet the challenges of human parturition. First, the relatively soft, unfused cranial bones of the infant slide and overlap during childbirth, essentially contracting the infant skull [61, 62]. The mother also produces a hormone called relaxin that softens the ligaments anchoring the pelvis, allowing it to stretch during delivery [63]. It has also been hypothesized that humans evolved a social system of support during childbirth to enable safer delivery of an infant born in the occiput-anterior position [17, 39, 64]. More critically, human infants are thought to be born at a ‘secondarily altricial’ stage of development compared to our closest primate relatives, leaving 70% of brain growth to be completed ex-utero [31, 65]. This has led to the characterization of the mother–neonatal dyad as completing its maturation after birth [65].

Notwithstanding the contentions surrounding these supposed adaptations, childbirth and especially deliveries for primiparous mothers can be a painful and protracted process with risks to both mother and infant, although humans are by no means exceptional in the degree of difficulty associated with labor [18, 29]. In countries with poor or no obstetrical care, or where there is unequal access to health care and/or where maternal health is generally low [66, 67], rates of morbidity and mortality are high [68], with potential complications of dystocia [69], uterine rupture [70], postpartum hemorrhage [71], sepsis [72, 73], fistulas [74] and other problems [75].

## HABITUAL SQUATTING AND EASE OF PARTURITION

In this extended commentary, however, we argue that childbirth was likely much easier for women in societies where squatting was *routinely* practiced by both females and males from an early age compared to contemporary lifestyles. We suggest that regular squatting significantly improves flexion at the sacro-iliac joint and enlarges the pelvic outlet allowing, for mothers, an easier passage of an infant through the birth canal.

### Mobility of the sacro-iliac joint

Many anatomists posit that ‘normal’ flexion of the sacrum within the pelvis is limited [54, 76]. Bogduk [76, pg. 170] stated flexion was <2° (i.e. the sacrum is virtually immovable); more recently, Gray’s Anatomy (pg. 1355) has similarly referred to this movement as <2°, while the role of relaxin in late pregnancy and during parturition is recognized as a temporary adaptation [54]. A recent systematic review covering 20 papers that had examined movement of the sacro-iliac joint accords with the assessment of limited movement except in some post-operative cases [77]. All of these studies derived either from patients or fresh cadavers and none of them looked at individuals in the squatting position. It is also extremely unlikely that any of the study participants squatted regularly. Effectively, the adult pelvis has three joints (Fig. 1): at the front is the cartilaginous pubic symphysis, which can be viewed as a pin joint in engineering terms, while the other two are the synovial sacro-iliac joints shaped as an ‘L’ around the interosseous sacro-iliac ligament that constitutes the primary bond between the sacrum and ilium [54, pg. 1354].

**Figure 2.**
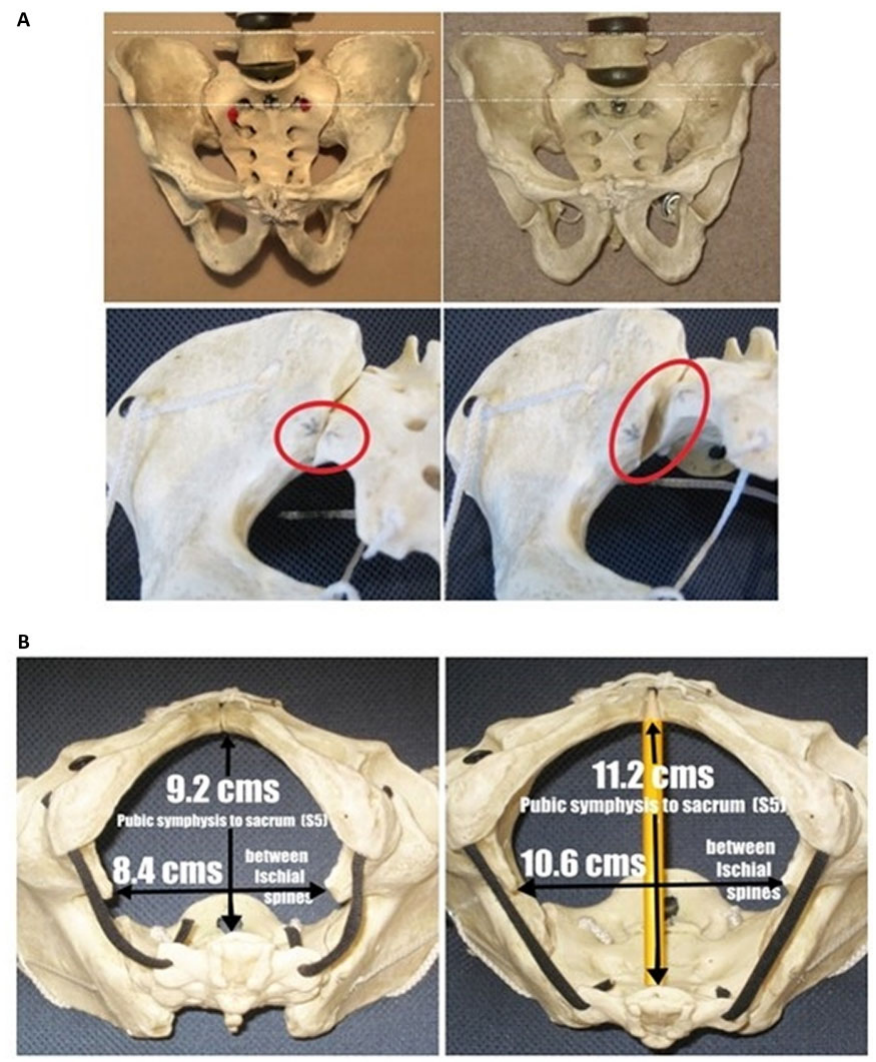
(**A**) Anterior and posterior views of a symmetrical pelvis (left) and the twist that occurs walking or running (right). The interosseous sacro-iliac (SI) ligament is simulated with a strong nylon cord that can be pulled tight. Top right illustrates the pelvis with the left foot forward as in the walking or running stride with the small twist that occurs between the ilia. Lower right shows the separation of the inferior leg of the SI joint that must occur for the S/I joint to allow this twist about the interosseous sacro-iliac ligament. (SI joints circled. Note the pencil arrow heads.) This requires a flexion of this SI joint of 25–30°. In a non-squatting lifestyle, the tight sacro-tuberous ligaments do not allow this flexion to occur simultaneously at both SI joints. (**B**) Inferior views of the pelvic outlet illustrating (left) ‘normal’ dimensions (i.e. without flexing the sacrum at the SI joints) and dimensions when both SI joints flex simultaneously (right)

As this strong sacro-iliac ligament is tight and inelastic, it remains the most important structure in the sacro-iliac joint but will still allow a ‘twist’ between the two sides of the pelvis and flexion of the sacrum between the ilia. This can be demonstrated if an individual lies supine with the iliac crests level and symmetrical to each other, while the two anterior superior iliac spines are also level (i.e. symmetrical). If the two ilia are maneuvered to twist in opposite directions, for example, by an experienced chiropractor, there should be a difference of at least 1–2 cm in the level of the two anterior-superior iliac spines, with one superior to the other (Fig. 2A). In the healthy and loose pelvis, this movement can be easily reversed with the other spine becoming superior. This movement belies the assertions by anatomists of minimal sacro-iliac mobility. The history of how the sacro-iliac joints came to be viewed as immobile is reviewed by Vleeming *et al*. who also argue in their comprehensive review that this viewpoint is erroneous [78].

The degree of flexion at the sacro-iliac joint is not easy to understand but, for the purposes of this commentary, is demonstrated using a model pelvis (see video, Supplementary Materials). This flexion will, of course, be limited by the muscles and ligaments of the pelvic floor, including the very strong sacro-tuberous ligaments. However, all of these muscles and ligaments can adapt their length according to a person’s lifestyle. The video, regardless of the absence of these ligaments and muscles in the model pelvis, explains how sacro-iliac flexion is compatible with the ridged and uneven surface of the sacro-iliac joints which is, otherwise, usually taken to confirm the limited mobility common to our contemporary lifestyle.

Figure 2B (left) illustrates what are considered to be the ‘normal’ dimensions of the pelvic outlet (i.e. without flexing the sacrum at the sacro-iliac joints). Even with the interosseous sacro-iliac ligaments held tight, the sacrum flexes easily within the two ilia and the shape of the sacro-iliac joint surfaces also increases the lateral ‘diameter’ of the pelvic outlet (between the spines of the ischia). Seen from the left, the sacrum rotates anti-clockwise, the center of rotation being the interosseous sacro-iliac ligament. *This ligament does not need to stretch to allow this*. The sacro-iliac joint surfaces are shaped such that the flexion of the sacrum causes a simultaneous lateral movement of the base of each ischium. We argue that there can be at least 20° rotation at both sacroiliac joints in a healthy pelvis where the sacro-tuberous ligaments have been stretched through appropriate activities such as squatting. Although the sacro-iliac joint is a synovial one, the inferior leg of the joint functions primarily as an ‘end-stop’ to prevent hyperextension of the joint.

Looking at the ‘ridged’, irregular (auricular) surfaces of the sacro-iliac joints (Supplementary Fig. 2), and the reported mobility of <2° of these joints in the literature, it is not surprising that many have concluded they have little to no mobility as discussed by Vleeming *et al*. [78]. The auricular surface ridges develop in early adulthood through the third decade and permit structural rigidity along the joint and interlocking facets [78, 79]; the joint surface has also been used to age adult skeletons in bioarchaeology [80]. The sacral surface of the sacro-iliac joint is concave and covered in hyaline cartilage and can be affected by osteoarthritis (OA), while the convex iliac surface is covered with thin fibrocartilage. The sacro-iliac joint is distinguished from other synovial joints by the unusual presence of these two different types of cartilage. Fibrocartilage is rigid and contains many collagen fibers, while hyaline cartilage is softer and contains fewer fibers. The auricular surfaces act as stress relievers or shock absorbers in activities such as running or jumping. However, if any part of the synovial surface was in a horizontal (coronal) plane, which is the ideal for static weight bearing, it would be liable to fracture during such stressful activities and would require much more robust bones as a consequence.

### Squatting and flexion

**Figure 3.**
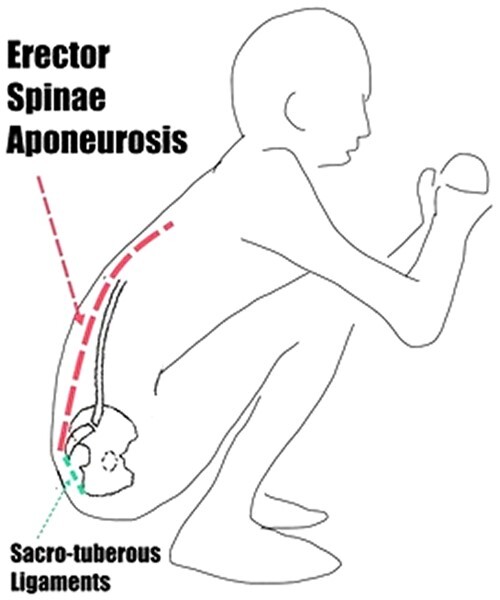
Forces exerted during squatting. Figure drawn by Yvonne Gorman

The kind of flexion of the sacrum demonstrated in Fig. 2 (and Supplementary Video) is negligible in our lifestyle. *Regular* squatting, however, is an activity that can both restore and maintain flexion. Squatting is defined as ‘a resting postural complex that involves hyperflexion at the hip and knee and hyperdorsiflexion at the ankle and subtalar joints’ [81, pg. 287] (Fig. 3). In squatting, the body weight is well forward of the hip joint, placing the force from the leg muscles onto the ilia and rotating them backwards. Simultaneously, the muscles of the mid and upper back need to be more active than usual in a seated position because the body is angled forwards instead of being vertical. This means that the erector spinae aponeurosis and other spinal muscles apply force to the sacrum in the opposite direction. The back muscles connected to the erector spinae aponeurosis thus provide support during squatting and have most of their inferior origin on the sacrum, tending to flex the sacrum within the pelvis. This is in the opposite direction to the forces on the ilia from the leg muscles, enabling squatting to provide a continuous force to stretch the sacro-tuberous and sacro-spinous ligaments and increase the flexion of the sacrum within the pelvis. The flexion force on the two sacro-iliac joints during squatting is large, and it is reasonable to assume that the mobility of the sacro-iliac joints in flexion (flexion of the sacrum between the ilia) is consistently greater among those living a lifestyle involving squatting, potentially for hours during the day.

Regular squatting has enormous implications for childbirth. Even temporary squatting can substantially increase the width of the pelvic outlet [77, 82–84], and evidence exists that this posture was likely preferred during parturition before the advent of modern obstetrical practices advocated women be supine [38, 60, 85]. Results from studies of squatting during childbirth in systematic reviews and meta-analyses are mixed, however, with low quality inferred for many of the contributing studies [86, 87]. Most studies are limited to squatting during the second stage of labor among women with little experience of this posture, and there is a lack of information provided on other confounds that might affect the progress and outcome of parturition, including anthropometric data and information on socioeconomic status.

In some parts of the world today, squatting is still a habitual posture, for example, during work with people placing their heels flat on the ground [88]. In some societies, the posture is used intermittently in yoga practice and is recommended during pregnancy [89]. Regular squatting is almost unknown in higher income countries and most adults living in affluent countries cannot squat with their heels on the ground. Michel *et al*. [83] noted in their paper that women became ‘exhausted’ by squatting within 10 min (pg. 1067), while an earlier intervention study in the UK resulted in a very small proportion of women being able to sustain squatting during the second stage of labor despite physiotherapy classes being held before birth to facilitate the posture [90].

## BIOARCHAEOLOGICAL EVIDENCE FOR SQUATTING IN THE PAST

**Figure 4.**
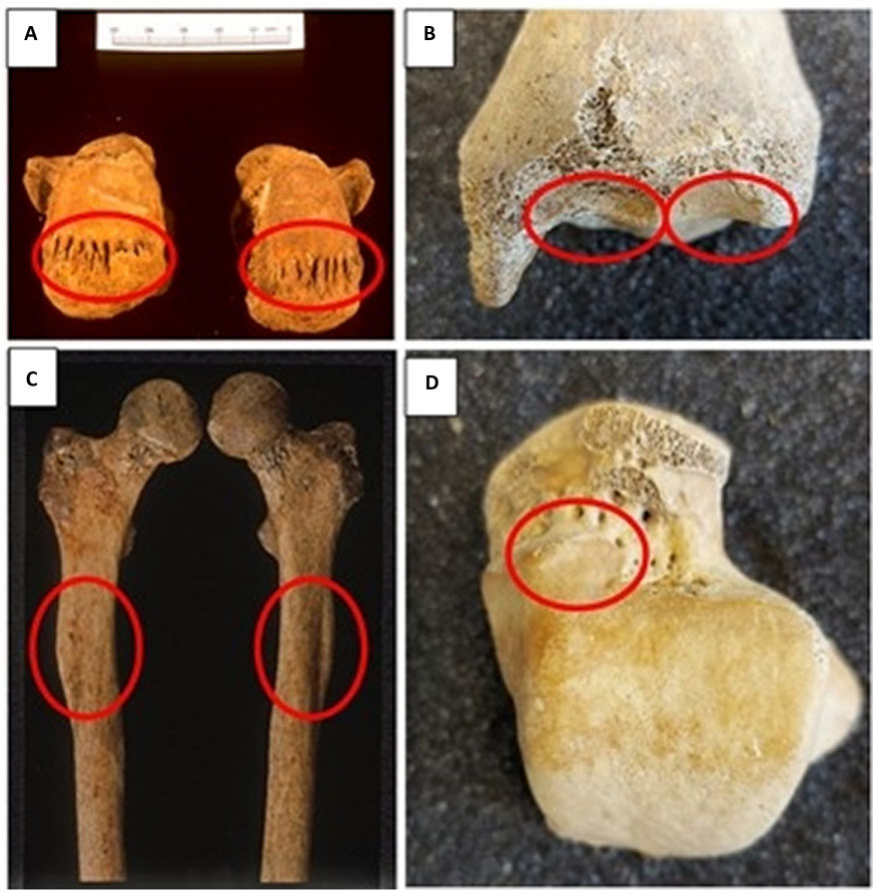
Actual (AC) and potential (P) osteological markers of squatting. (**A**): New bone formation at achilles tendon attachment to posterior aspects of the calcanea (P). (**B**): Morphological changes to the anterior distal end of the tibia (ankle joint)—medial and lateral squatting facets (AC). (**C**): Antero-posterior flattening of the proximal femoral shafts (platymeria) (P) (**D**): Morphological changes to the neck of the talus—medial extension of the articular surface (AC).

Data from multiple sources (e.g. ethnographic, archaeological, palaeoanthropological, palaeopathological) suggest humans have squatted during the majority of our bipedal existence, thus strengthening our pelvic ligaments and muscles. Tuttle *et al*. [91] outlined habitual squatting in many closely related non-human primates, possibly supporting deep ancestral roots to this posture [cf. 92]. Habitual dorsiflexion elongates the ankle ligaments, and can lead to morphological changes to the lower leg and foot bones, including stress on the achilles tendon that attaches to the calcaneum (Fig. 4). Other anomalies in the bones of the skeleton, usually defined as normal variation and referred to as non-metric traits, are described as having a genetic basis and/or linked to physical activity/biomechanical loading, or certain postures including squatting [93–95]. These include: (i) retroversion of the tibia, described as a ‘backward incline of the tibial plateau’ (pg. 50) [96]; (ii) the Poirier’s facet on the proximal end of the femur (lateral expansion of the anterior part of the femur head toward the anterior aspect of the femoral neck) [93, 97]; (iii) plaque formation (imprint on the anterior margin of the femoral neck close to the femur head) [98]; and (iv) the Allen’s cervical fossa/cribra/reaction area [97, cf. 99].

However, there is evident confusion in correctly attributing the presence or absence of the individual femoral neck traits [99]. Some research has shown that the average declination angle of the tibial plateau in people who regularly squat is 14–17° but, for European populations, the angle is less [96, 100]. Furthermore, in a study of fetal skeletons, the average angle was reported to be 19.8° (caused by constant flexion of the knees during the fetal period), which may explain findings of higher rates of squatting facets in fetal skeletons compared to adults [96]. Reduction of this flexion post-natally leads to straightening of the head of the tibia and any tibial retroversion is therefore not considered a reliable indicator of squatting [96, 101].

### Squatting facets

Other skeletal traits are explicitly called ‘squatting facets’ caused by pressure of the anterior edge of the distal tibia on the neck of the talus. They may be recognized as medial and lateral expansions of the distal tibial articular surface onto the anterior aspect of the metaphysis, and on the medial and lateral sides of the neck of the talus (Fig. 4B and D). Such facets have been related to the squatting posture where flexion of the foot places the talar neck proximal to the distal tibia [96], and are documented in ancestral skeletons, including Neanderthals, whom Erik Trinkaus [102] described as ‘habitual squatters’ (pg. 346).

In a study of the lateral squatting facet in tali and retroversion in the tibiae of skeletons dating from the 1st to 20th centuries AD, mainly from France, Boulle [96] concluded that squatting was regularly adopted as a posture until the end of the Middle Ages (late 15th/early 16th centuries AD). This corresponded with a change in the organization of interior household space and the introduction of stoves and furniture, such as seating, that may have altered domestic habits and postures. This could have led to less squatting and hyperdorsiflexion of the ankle, in favor of standing and sitting, as would later intensification and mechanization of agriculture. Furthermore, in European skeletons, some studies show that women had more squatting facets than men, which suggests a division of tasks linked to the need to squat [103]. Mays further highlights the lower frequency of squatting facets among higher status people interred in the 18th/19th century crypt at Christchurch, Spitalfields, London, compared to the higher frequency in lower status people buried in the cemetery associated with rural Wharram Percy, North Yorkshire, England [103]. Here, both men and women would have been involved in activities related to farming that could have required squatting, but more women than men had squatting facets.

In another study in South Africa, Dlamini and Morris [104] found that half of the skeletons (28 of 56) of Late Stone Age foragers (first millennium BC) were habitual squatters, the majority (13 of 17) of 5–19th century farmers, and only 1 of 21 18th century ‘Free Blacks’ and/or enslaved individuals from the Cobern Street skeletal collection had squatting facets. In this study, the availability or lack of furniture for sitting on, as well as cultural and individual differences in posture, were provided as interpretations for the results. Dewar and Pfeiffer [105] also found most of the c.100 skeletons dated to later Stone Age Africa had evidence of squatting facets.

### Platymeria and platycnemia

Two other osteological features have been connected to squatting, namely platymeria and platycnemia. Platymeria (Fig. 4C) has been linked to a biomechanical adaptation derived from the squatting posture and is variable across populations, but Brothwell [106] expressed the opinion that the ‘jury was [still] out’ (pg. 90), suggesting that it may be a ‘non-metrical variation of a non-environmentally-induced kind’. It is described as the antero-posterior flattening of the proximal end of the shaft of the femur, assessed by calculating the femur shaft index taking the antero-posterior and medio-lateral diameters just below the lesser trochanter (Fig. 4). A platymeric femur is described as one with an index of <84.9 while an eurymeric femur has an index of 85–99.9 [106]. Lower platymeric indices ‘tend to be associated with the entire range of preagricultural hominids, fossil and extant’ (pg. 381) [107]. Indeed, increased platymeric indices are associated with people who have lived their lives in an agricultural world. Therefore, squatting in pre-agricultural foraging communities could have contributed to the occurrence of platymeria, and a reduction in physical activity in farming would have led to higher indices. In a study of skeletons from Greece over time (Neolithic to Byzantine), Angel [108] also found that platymeria declined over time.

In contrast, platycnemia is characterized by medio-lateral flattening of the proximal end of the tibia, assessed by taking the antero-posterior and medio-lateral diameters at the level of the nutrient foramen on the posterior aspect of the tibial shaft. In discussing platymeria and platycnemia, Buxton [109] referred to various causes of the flattening of these bones being related to ‘posture, gait, or both’ (pg. 31) including squatting, or to such activities as mountain climbing. Charles [110] had linked squatting to both platycnemia and squatting facets, a hypothesis with which Cameron [111] agreed. The latter had already dismissed squatting as a cause of platymeria because it was absent from ‘Eskimo’ (Inuit) populations who were thought to have squatted, although Cameron did think that this posture caused platycnemia and tibial retroversion [see also 112]. Hagihara and Nara [113] further noted that platycnemia has been reported in the tibiae of Jomon hunter-gatherers from Japan and likely reflects lower limb loading. Nevertheless, correlation does not equal causation.

The retroauricular area of the ilium, already discussed above, and its soft tissue attachments will stretch (lengthen) in the squatting posture. Both squatting and platycnemia have also been linked to OA of the joints (including the sacro-iliac joint), but of course increased age would likely contribute to OA, alongside posture/lifestyle associated with squatting. Therefore, might we expect to find squatting facets and other associated markers like platymeria and platycnemia in individual skeletons, along with OA of the sacro-iliac joint? The preauricular sulcus (Supplementary Fig. 2) has also been associated with pregnancy/childbirth, although this continues to be debated [114–117]. Recently, Igarashi *et al*. [118] have argued that parturition scars can at least be related qualitatively to nulliparity, as well as to low and high fertility. The problem is that specific types of scars (e.g. the preauricular sulcus) can be found in biological males, and it is suggested that there are male and female types of scars at this anatomical location.

Cameron [111] further suggested that poor nutrition could be a potential cause of femoral and tibial flattening. Brickley *et al*. [119] also mentioned a relationship between platymeria and vitamin D deficiency in adult skeletons buried in 18th and 19th century Birmingham, England, during the Industrial Revolution, suggesting that related deformities were connected to weight-bearing on weak and poorly mineralized bone. Martin *et al*. [120] had previously reported that bone mineral content decreased in the tibiae of female Alaskans by 50% between the third and sixth decades, but not in males. However, based on comparative studies of health, pre-agricultural prehistoric populations would likely not be nutritionally deficient or lacking in exposure to ultraviolet light (the main cause of vitamin D deficiency).

In general, the health of people appears to have declined over time and with the introduction of farming as the main subsistence economy. The Neolithic Revolution led to a reduction in dietary diversity for many, at least in the Middle East and Europe, with several bioarchaeological studies revealing nutritional deficiencies as well as decreases in stature [121–124]. In other parts of the world, such as southeast Asia, people may have fared better at the agricultural transition [125]. Archaeological data from agricultural and later sites show differences in pelvic dimensions between low and high status individuals or generally show evidence for poor nutritional status of individuals with flatter pelves [126].

Women with reduced stature are known from contemporary obstetrical studies to have more complicated births [36–38], and are at higher risk for flattened pelves and obstructed deliveries [36, 39]. This is especially the case if women are younger primipari whose growth is not yet complete, introducing trade-offs between growth, maintenance and reproduction [34]. Such trade-offs possibly became more prominent with agriculture and an increasing prevalence of nutritional inadequacies exacerbated by growing social inequalities [35, 127]. It is therefore possible that the Neolithic period heralded the start of more complicated births for humans that had more to do with ecological than genetic constraints. In addition, domesticated crops such as wheat, rice and corn may have provided suitable weaning foods such as paps that could have shortened periods of lactation and reduced post-partum amenorrhea and inter-birth intervals, thereby increasing risks for successive parturition, maternal depletion and poor maternal health.

One aspect of Neolithic sedentism that likely affected the physiology of settlers with consequences for childbirth was an increasing use of seats (most likely stools in the beginning) that would have replaced the more traditional method of squatting. Of course, individuals could have used logs or large stones rather than specially crafted seats. Hunter-gatherers likely also sat on the ground for various activities such as flint-knapping. Since the first stools may have been made of wood, their survival is unlikely in the prehistoric record but probably predate any depictions accidentally discovered at archaeological sites. Already by 8000 ya at the Neolithic side of Çatalhöyük in Turkey, complex chairs (at least for the elite) were being made judging by the discovery of a pottery figurine depicting a large woman seated on an elaborate throne-like structure [128]. Two small ceramic figurines, one male and one female, each sitting on a stool, were also found at the Neolithic site of Cernavoda in Romania dating to around 7000 ya, and at another Romanian site at Târpeşti, a figurine sitting on a chair was also discovered. The ceramic Gilat Woman found at the Chalcolithic site of Gilat in Israel dating to 6000 kya is seated on a conical stool [129]. It is of course unknown how widely available such items would have been to the general population at such sites or if they were considered luxuries.

## CONCLUSIONS AND IMPLICATIONS

In this extended commentary, we have constructed a picture of potentially easier childbirth for ancestral women who likely squatted regularly. This argument obviates hypotheses explaining trade-offs between a small human pelvis relative to a large infant skull, arguing that habitual squatting would have considerably facilitated the birth process. Using a biomechanical model, we have demonstrated how squatting can enlarge the pelvic outlet diameter by almost 2.5 cm in all people (females and males) and possibly by much more in the latter stages of pregnancy if the hormonal changes enable additional stretching of the very strong sacro-tuberous ligament to increase degrees of flexion at the sacro-iliac joint. We considered evidence from the bioarchaeological and ethnographic record for squatting, a posture still used in many communities where lifestyles facilitate its practise. Earlier ethnographic data consistently referred to easier births among women within such communities.

It may be possible to test this ‘Habitual Squatting Hypothesis’ further by examining individuals who regularly squat to examine their pelvic flexion, ideally under an MRI machine similar to studies already cited above on squatting postures in women [83, 84]. However, access to MRIs would not be possible for many such individuals in the requisite settings. Another suggestion would be to run interventions among young women by introducing them to squatting exercises and encouraging them to perform every day activities (where possible) in the squatting position. Of course, compliance with such an exercise program would be difficult to monitor appropriately and would require highly motivated participants, but this might be possible if the benefits were fully appreciated and if introduced early enough through school programs.

## Supplementary data

Supplementary data is available at *EMPH* online.

## AUTHORS’ CONTRIBUTIONS

J.G. designed the conceptual model; all authors contributed to writing the paper.

**Conflict of interest:** None declared.

## DATA AVAILABILITY

All data are included in the paper and supplementary materials.

## Supplementary Material

eoac017_Supplementary_Data
