# Supplementary material for: Squatting, pelvic morphology and a reconsideration of childbirth difficulties
Source: Evol Med Public Health. 2022 Apr 26;10(1):243–55. doi: 10.1093/emph/eoac017 (PMC9154243; doi:10.1093/emph/eoac017)
Supplement: eoac017_Supplementary_Data [file eoac017_supplementary_data.zip › GormanetalSupplementaryVideoLink.docx]

**Supplementary Video File for:**

**Squatting, Pelvic Morphology, and a Reconsideration of Human Childbirth**

Running title: **Squatting and human childbirth**

John Gorman, Charlotte A Roberts, Sally Newsham, Gillian R Bentley

**This PDF file includes:**

Supplementary video that explains the osteological model based on engineering principles and evidence from physical therapies.

<https://www.dropbox.com/s/tgzhr05zruyrmca/Lecture%20with%20squatting.15th%20April.mp4?dl=0>
