# Supplementary material for: Squatting, pelvic morphology and a reconsideration of childbirth difficulties
Source: Evol Med Public Health. 2022 Apr 26;10(1):243–55. doi: 10.1093/emph/eoac017 (PMC9154243; doi:10.1093/emph/eoac017)
Supplement: eoac017_Supplementary_Data [file eoac017_supplementary_data.zip › GormanetalSupplementaryFigures.docx]

**Gorman et al. (2022) Squatting, Pelvic Morphology, and a Reconsideration of Childbirth Difficulties. Supplementary Figures**

Supplementary Figure 1. “Racial typologies” of pelvic shapes constructed in the early twentieth century and prominent even today in obstetric and midwifery textbooks. Redrawn by Yvonne Gorman.

Supplementary Figure 2. View of the ilium showing auricular surfaces and preauricular sulcus.
